# The Effect of Accessibility of Insoluble Substrate on the Overall Kinetics of Enzymatic Degradation

**DOI:** 10.1002/bit.28921

**Published:** 2025-01-06

**Authors:** Zdeněk Petrášek, Bernd Nidetzky

**Affiliations:** ^1^ Institute of Biotechnology and Biochemical Engineering Graz University of Technology Graz Austria; ^2^ Austrian Centre of Industrial Biotechnology Graz Austria

**Keywords:** cellulose, enzyme kinetics, insoluble substrate, modeling, substrate accessibility, surface area

## Abstract

The enzymatic reaction kinetics on cellulose and other solid substrates is limited by the access of the enzyme to the reactive substrate sites. We introduce a general model in which the reaction rate is determined by the active surface area, and the resulting kinetics consequently reflects the evolving relationship between the exposed substrate surface and the remaining substrate volume. Two factors influencing the overall surface‐to‐volume ratio are considered: the shape of the substrate particles, characterized by a single numerical parameter related to its dimensionality, and the distribution of the particle sizes. The model is formulated in a form of simple analytical equations, enabling fast and efficient application to experimental data, and facilitating its incorporation into more detailed and complex models. The application of the introduced formalism exploring its potential to account for the observed reaction rate is demonstrated on two examples: the derivation of particle size distribution from experimentally determined reaction kinetics, and the prediction of reaction slowdown from experimental particle size distribution.

## Graphical abstract

The evolution of the surface‐to‐volume ratio of the substrate particle influences the observed reaction kinetics. The surface area may increase (cylindrical pore: blue) or decrease (sphere: red, a particle with a structured surface: green) as the reaction proceeds. Substrate with a broad distribution of particle sizes is analogous to a particle with a structured surface.

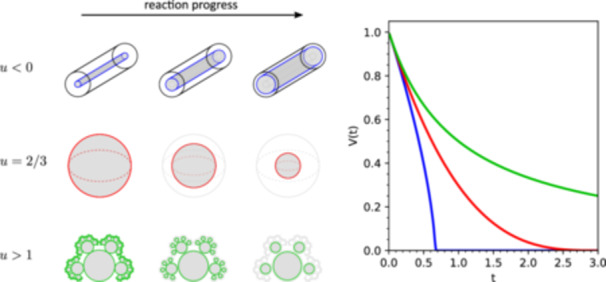

## 1 Introduction

Cellulose is an abundant natural material that can be utilized to produce a range of soluble carbohydrate products. It is available in a form of a solid substrate where the polymeric carbohydrate chains are hierarchically arranged, with a variable degree of regularity, resulting in an insoluble material (Chundawat et al. 2011). The natural cellulosic material is typically subjected to a pretreatment resulting in water‐insoluble particles with sizes ranging from nanometers to millimeters, before it undergoes enzymatic depolymerization (Hendriks and Zeeman 2009).

A major problem for the utilization of cellulose is the often observed slowdown of the enzymatic hydrolysis in the course of the reaction (Bansal et al. 2012; Desai and Converse 1997; Väljamäe et al. 1998; Yang, Willies, and Wyman 2006; Jalak and Väljamäe 2010). A short initial phase of fast conversion (hydrolytic burst) is followed by a period of gradually decreasing reaction rate, for some substrates coming to a halt before all substrate is converted (Cruys‐Bagger et al. 2012; Murphy et al. 2012; Praestgaard et al. 2011). The origin of this behavior can be traced back to the insolubility of the particulate substrate, which limits the accessibility of the substrate to the enzyme and enables undesirable interactions of the enzyme with the substrate particles, such as nonproductive binding (Väljamäe et al. 1998; Kurašin et al. 2015). Other related factors include crowding and blocking of the enzyme molecules bound to the substrate surface (Väljamäe et al. 1998; Payne et al. 2015; Igarashi et al. 2011), reversible or irreversible adsorption on noncellulosic components, such as lignin (Palonen et al. 2004; Berlin et al. 2005), and the complex substrate morphology and heterogeneity evolving in the course of degradation (Nill and Jeoh 2020; Peciulyte et al. 2015; Ganner et al. 2012).

Among the mentioned effects, the substrate accessibility is a universal factor that can be expected to affect any enzymatic reaction on an insoluble substrate, regardless of the details of the enzymatic reaction mechanism and the type of material under attack. Only the reactive substrate sites exposed to the solution can be attacked by the enzyme. These include the sites not only on the outer surface of the solid substrate particles, but also on the inner surfaces of any pores, voids or channels connected with the outside and large enough to allow penetration of enzyme molecules (Bansal et al. 2012; Grethlein 1985; Zhang and Lynd 2004; Bansal et al. 2009; Hong, Ye, and Zhang 2007; Zhao, Zhang, and Liu 2012).

The extent of substrate accessibility has also been implicated in degradation of other insoluble substrates. The digestion of native (not gelatinized) starch granules exhibits two phases, fast and slow (Dona et al. 2010; MacGregor and Ballance 1980; Meraz et al. 2020; Oates 1997), and depends on the granule surface area, in addition to their composition and internal structure. The enzymatic activity is better described as a function of the surface area than of the substrate concentration (Kong et al. 2003), with smaller granules having larger specific surface area and therefore being digested faster than the large ones (Qi and Tester 2016). Similarly, the complex supramolecular structure (crystallinity) of chitin limits the efficiency of its degradation by enzymes. As with cellulose, different methods of pretreatment are used to increase the accessibility of chitin to the enzyme (Li et al. 2019; Zhang et al. 2018; Chen et al. 2015). Another area where substrate accessibility plays a crucial role is biodegradation of plastics by enzymes exploited either as an alternative to mechanical recycling, or as a source of valuable depolymerization products (Tournier et al. 2023; Ellis et al. 2021; Cai et al. 2023; Taniguchi et al. 2019). Both synthetic plastics and bioplastics share some relevant characteristics with the natural biological substrates mentioned above: they are polymeric, insoluble in water, and exhibit a wide variation in morphology. In particular, the size of the plastics particles and their surface structure were shown to influence the rate of plastics degradation by enzymes (Hino et al. 2023; Brizendine et al. 2022; Weinberger et al. 2017).

To understand the role of substrate accessibility, the limitations imposed by insoluble substrate are sometimes incorporated into models of cellulose degradation. The reaction rate is affected by the accessible surface area, which changes in the course of the reaction both in absolute terms and in relation to the remaining substrate volume. The exact relationship between the active surface area and the substrate volume in these surface ablation models (Jeoh et al. 2017) depends on the particle shape. The most commonly modeled shape is a cylinder (Zhou et al. 2009a; Griggs, Stickel, and Lischeske 2012a; Zhang, Xu, and Zhou 2014; Huron et al. 2016; Movagarnejad et al. 2000; Piątek, Lisowski, and Dąbrowska 2021), based on the filamentous structure of cellulose; in other cases the particles were assumed to be spherical (Zhou et al. 2009a; Piątek, Lisowski, and Dąbrowska 2021; Levine et al. 2010) or to have a constant surface area (Zhou et al. 2009a). Some models also included pores or cavities inside the substrate particles. These were modeled either as slits, that is, with a constant surface area (Luterbacher, Parlange, and Walker 2013; Rohrbach and Luterbacher 2021), or as having cylindrical or spherical shape (Hobson 1987).

The overall surface to volume relation of the substrate is, however, determined not only by the particle shape but also by the size of the substrate particles. The often used assumption that all substrate particles have the same initial size is rather restrictive. Therefore, in a few cases, the distribution of particle sizes has been included in the characterization of the substrate. In one model two discrete sizes of spherical particles were considered (Levine et al. 2010). Other models assumed a broad uniform (Zhou et al. 2009b) or a gaussian (Zhang, Xu, and Zhou 2014; Zhou et al. 2009b) distribution of cylindrical particles. In another case, an experimental distribution was combined with a spherical particle shape (Sanders et al. 2000).

In addition to the effects of evolving active surface area, the theoretical models of cellulose hydrolysis usually contain a range of other mechanistic details that shape the reaction kinetics. The model features include the details of the substrate structure, the enzyme properties and the details of the enzyme‐substrate interactions (Bansal et al. 2009). Different types of substrate surface sites have been modeled (Zhou et al. 2009a; Zhang, Xu, and Zhou 2014; Eibinger et al. 2016) as well as the cellulose chain length distribution and its temporal evolution (Zhou et al. 2009a; Griggs, Stickel, and Lischeske 2012a; Zhang, Xu, and Zhou 2014; Huron et al. 2016; Levine et al. 2010). Several models include simultaneous action of different enzymes (Zhou et al. 2009a; Huron et al. 2016), enzyme inhibition (Movagarnejad et al. 2000; Levine et al. 2010; Griggs, Stickel, and Lischeske 2012b) or enzyme deactivation (Huron et al. 2016; Movagarnejad et al. 2000). The enzyme interaction with the substrate has been resolved into more reaction steps (such as initial attachment, threading, de‐threading, etc.) (Griggs, Stickel, and Lischeske 2012a; Levine et al. 2010), other models included blocking of binding sites due to crowding (Levine et al. 2010).

Although these comprehensive models aim at reproducing the degradation kinetics as faithfully as possible, due to their complexity the actual contribution to the kinetics of the individual model features is is usually not evaluated. Additionally, analytical solutions of the complex models are typically not feasible, and the models normally have to be solved numerically. This further limits a deeper understanding of the effect of the composition of the model, such as the role of the substrate accessibility, on the resulting behavior.

Here we consider the effects on the reaction kinetics of the particle shape and particle size distribution isolated from other factors, and explore their potential to account for the observed reaction slowdown. The aim is to introduce a general formalism that includes both the particle shape and the particle size distribution, and that leads to easy‐to‐use analytical solutions suitable for practical description and analysis of experimental reaction kinetics. Further, we test the applicability of the introduced framework on two complementary tasks: to obtain a particle size distribution that would result in an observed reaction rate slowdown, and, conversely, to calculate the expected slowdown given an experimental particle size distribution.

## 2 Results and Discussion

### 2.1 Reaction Rate

The substrate is present in a form of solid particles suspended in solution. It is assumed that the reaction takes place only on the surface of the substrate particles, that is, the amount of the substrate available for the reaction at any time is directly proportional to the accessible surface area. The rate of the reaction is therefore a function of the accessible surface area A, and not directly the total amount of the substrate, which we will specify by its volume V:

(1) $$\frac{\mathrm{dV}}{\mathrm{dt}}=-f(A(V)).$$

We assume that the reaction rate is proportional to the concentration of the complexes between the enzyme and the reactive binding sites on the substrate surface. The concentration of these complexes C is described by the binding equilibrium with the equilibrium constant K: $K=\mathrm{ES}/C=(E_{0}-C)(S_{0}-C)/C$, where $E_{0}$ and E are the total and the free (not bound) enzyme concentrations, respectively, and $S_{0}$ and S are the concentrations of all and unoccupied substrate surface binding sites, respectively.

In a general situation, the equilibrium condition can be reformulated to a Langmuir equation $C=S_{0}E/(K+E)$, or, when expressed using $E_{0}$ and $S_{0}$, C can be calculated as a root of a quadratic equation: $C=(K+E_{0}+S_{0}-\sqrt{{\left(K+E_{0}+S_{0}\right)}^{2}-4E_{0}S_{0}})/2$. This implies a rather complex dependence of the reaction rate f(A) (Equation 1) on the concentration of the surface binding sites $S_{0}$, that is, on the surface area A.

In the limit of excess enzyme, $E_{0}\gg S_{0}$, the formula for C can be simplified to $C=S_{0}E_{0}/(K+E_{0})$, with the concentration of complexes C being proportional to the concentration of the surface binding sites $S_{0}$. In other words, the reaction rate is directly proportional to surface area A: f(A)=kA, where k is a constant, which leads to:

(2) $$\frac{\mathrm{dV}}{\mathrm{dt}}=-\mathrm{kA}.$$

This implies that the number of the surface binding sites on the substrate is the rate limiting factor. Such situation is known from cellulose hydrolysis by cellulases (Kari et al. 2017; Lynd et al. 2002; Bader et al. 1992; Ooshima, Burns, and Converse 1990; South, Hogsett, and Lynd 1995) when the enzyme concentration may be higher than the concentration of the accessible substrate sites.

When this approximation does not hold, other descriptions of the dependence of the reaction rate on the surface area can be employed. For example, in the opposite situation of the excess of the binding sites, $S_{0}\gg E_{0}$, one obtains $C=E_{0}S_{0}/(K+S_{0})$, resulting in the rate f(A)=kA/(K+A). When the binding is strong (small K) the complex concentration C can be further simplified to $C\approx E_{0}$, giving f(A)=k, that is, all enzyme is bound to the substrate, and the reaction rate is independent of the substrate surface area. In this work, however, we limit ourselves to the rate as specified in Equation 2, because we want to keep focus on the effects of the surface area only, not on the combination with effects of limited enzyme amount or the substrate‐enzyme binding affinity.

### 2.2 Particle Shape

The shape of the substrate particle defines the relationship between its volume V and its active surface area A. As shown below, a convenient and a sufficiently general way to describe the dependence of the surface area A on the volume V is:

(3) $$A(V)=aV^{u},$$

where we first assume that a and u are unspecified constants depending on the particle shape.

Substituting Equation 3 in Equation 2 results in the rate equation for the total substrate volume V:

(4) $$\frac{\mathrm{dV}}{\mathrm{dt}}=-\mathrm{ka}V^{u}.$$

This rate equation can be directly integrated, giving the solution as the decrease of the total substrate volume V(t) with time, given the initial volume $V_{0}$:

(5) $$u\neq 1:\;V(t)=V_{0}{\left(1-\mathrm{ka}(1-u)V_{0}^{u-1}t\right)}^{\frac{1}{1-u}}.$$

(6) $$u=1:\;V(t)=V_{0}e^{-\mathrm{kat}}.$$

**Figure 1:**
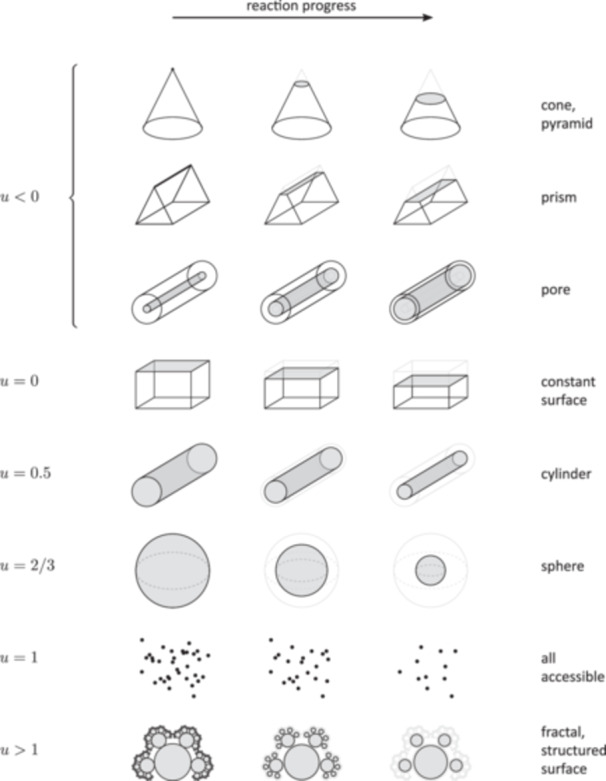
The classification of the particle shapes by the shape parameter u. The evolving active surface area (gray) relative to the remaining volume during the reaction is shown.

Apart from leading to a simple analytical solution, the description of the surface–volume dependence using Equation 3 and the parameter u is advantageous because it describes simple shapes straightforwardly: a sphere with u=2/3, a cylinder (without the reactive end surfaces, or long enough so that the end surfaces can be neglected): u=0.5, and a block with a constant reactive surface: u=0 (see also Figure 1).

When 0<u<1, it is convenient to introduce a parameter x, having the intuitive meaning of a coordinate perpendicular to the particle surface, a direction along which the particle is consumed. During a time interval dt the volume dV of the particle is consumed; this volume dV is proportional to the particle surface A and the thickness dx of the imagined consumed surface layer: dV=A dx. Using Equation 2 we get:

(7) $$\frac{\mathrm{dV}}{\mathrm{dt}}=\frac{\mathrm{dV}}{\mathrm{dx}}\frac{\mathrm{dx}}{\mathrm{dt}}=A\frac{\mathrm{dx}}{\mathrm{dt}}=-\mathrm{kA},\;\text{therefore}\;\frac{\mathrm{dx}}{\mathrm{dt}}=-k,$$

which results in:

(8) $$x(t)=x_{0}-\mathrm{kt},$$

in analogy to contraction models in solid‐state kinetics (Khawam and Flanagan 2006). Equation 8 thus confirms that x(t) can be interpreted as a coordinate with the direction along which the substrate is consumed (a generalized radius). Starting at $x_{0}$, the value of x decreases linearly with time as the substrate particle is being converted. The remaining particle volume is at any time equal to V(x):

(9) $$V(x)={\left(\frac{\mathrm{ax}}{d}\right)}^{d},$$

where d is an effective dimensionality describing the decomposition of the particle, and a is a constant. The expression for the volume of the common shapes has then the usual form: a sphere: $V(x)=4\pi x^{3}/3$, a cylinder (without reactive ends) with length h: $V(x)=\pi x^{2}h$, a slab with a constant surface $A_{0}$: $V(x)=A_{0}x$ (Table 1). The dimensionality d is directly related to the more general shape parameter u: u=1−1/d.

**Table 1:** The characteristics of different substrate particle shapes from the point of view of the evolving surface‐to‐volume relationship described by the shape parameter u. The proportionality constant a (Equation 3) and the temporal volume decrease V(t) are given for the three regular shapes with effective dimensionalities d=1 (slab), d=2 (cylinder) and d=3 (sphere). See also Figure 1.

u	d	a	V(t)	Shape characteristics
<0				Increasing active surface area; crystal
0	1	$A_{0}$	$x_{0}A_{0}\left(1-\frac{k}{x_{0}}t\right)$	Constant surface area $A_{0}$ (1D)
0.5	2	$2\sqrt{\pi h}$	$\pi x_{0}^{2}h{\left(1-\frac{k}{x_{0}}t\right)}^{2}$	Cylinder (2D)
2/3	3	${\left(4\pi \right)}^{1/3}3^{2/3}$	$\frac{4}{3}\pi x_{0}^{3}{\left(1-\frac{k}{x_{0}}t\right)}^{3}$	Sphere (3D)
1				Constant substrate fraction accessible
>1				fractal‐like shape; eroded sample

Using x(t) allows us to reformulate the solution given by Equation 5 into a simpler form:

(10) $$V(t)=V_{0}{\left(1-\frac{k}{x_{0}}t\right)}^{d}=V_{0}{\left(\frac{x(t)}{x_{0}}\right)}^{d}.$$

This is the main formula describing the kinetics of degradation of particles with a shape characterized by the parameter u between 0 and 1, or, alternatively, by the effective dimensionality d∈(1,∞).

The solution (Equation 5, 10) is valid only for the times $t<t_{e}$, where $t_{e}$ is the time point at which all substrate converted (for u<1). From the condition $V(t_{e})=0$ (or, alternatively, $x(t_{e})=0$) we obtain:

(11) $$t_{e}=\frac{V_{0}^{1-u}}{\mathrm{ka}(1-u)}=\frac{x_{0}}{k}.$$

**Figure 2:**
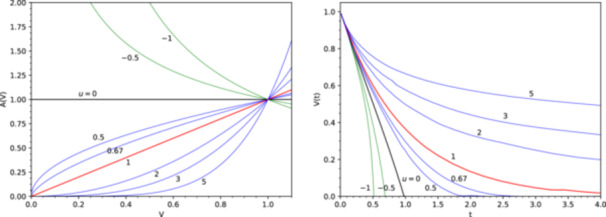
The relationship between the particle volume V, particle surface area A and the reaction time t for different shape parameters u in the range from ‐1 to 5. Left: the relative dependence of the surface area A on the particle volume V. Right: the relative decrease of the substrate particle volume V with the reaction time t.

The shape parameter u represents the different degrees of dependence of the particle surface area, and therefore the reaction rate, on the remaining particle volume (Figure 2).

The intermediate values between 0 and 1 can be used to effectively describe different particle geometries, with characteristics intermediate between the basic shapes shown in Table 1. At u=0 the surface area and the reaction rate are constant, and the substrate volume decreases linearly in time. With larger u, the surface area starts to decrease at a progressively larger rate as the volume decreases, and the reaction rate slows down as the reaction proceeds (Figure 2).

A special case is u=1, when the surface area and the reaction rate are proportional to the remaining substrate volume. This is equivalent to all the substrate, or a constant fraction of the remaining substrate, being accessible to the reaction, similarly to a general situation of a homogeneous solution of fully dissolved substrate, and leads to the usual first order reaction kinetics (Equation 6).

Not all shapes can be described by u that remains constant throughout the reaction. A cylinder with reactive end surfaces is described by u varying between 0 and 2/3. A special case is a cylinder with a diameter equal to its length ($p\equiv h_{0}/(2r_{0})=1$): from the point of view of reaction kinetics it behaves as a sphere: u is constant throughout the reaction and equal to 2/3. If the cylinder is elongated (p>1), the relative contribution of the reactive ends decreases as the reaction proceeds, and u converges to 0.5. If the cylinder is initially short, resembling a disc (p<1), the reactive ends increasingly dominate, and u converges to 0.

The case when u is negative corresponds to a substrate with increasing reactive surface area as its volume decreases (Figure 1). This can describe, for example, crystalline substrate, where initially only a crystal edge, or a single crystal face, is reactive. As the reaction proceeds, the surface area susceptible to the reaction grows. Cellulose crystals are an example of substrate structures with preferential enzyme binding sites located on a particular crystal plane (Lehtiö et al. 2003). Examples of shapes with this property include a triangular prism with initially only one edge being reactive (developing into an enlarging face), and a cone, where the conversion to product starts at its apex and proceeds on an enlarging circular surface as it moves towards the base of the cone (Figure 1). In these examples u is not constant during the reaction, but still stays negative at all times. Negative u describes also porous substrate particles digested from inside. During the reaction the internal particle surface area increases as the pores and cavities are enlarged (Figure 1). Pores and cavities are present in starch granules (Huber and BeMiller 1997; Villwock and BeMiller 2022) and are known to play an important role in their hydrolysis (Oates 1997).

Also the case of u>1, with an initial fast decline of the surface area (Figure 1), has a meaningful interpretation. It can serve as a model of a fractal‐like particle with highly structured or eroded surface with a lot of fine detail. Alternatively, it can model a lose aggregate of particles of different sizes, with smaller particles on the aggregate surface. The initial phase of the reaction is characterized by a high reaction rate and a fast decrease of the surface area while a relatively small fraction of the volume is converted. This phase is described by u>1. At later stages, the particle surface becomes increasingly smoothed‐out, the reaction rate decreases, and u may eventually decrease below 1.

In summary, the shape parameter u and Equation 3 provide a sufficiently general and unifying description of different substrate particle shapes. While for u=0 the reaction rate is constant, independent of the volume, for negative u the reaction rate (and the surface area) increases as the volume decreases, and for positive u the reaction rate decreases with the decreasing substrate volume.

### 2.3 Particle Size Distribution

The results in the previous section can be applied as a model for a single particle or for an ensemble of particles of the same initial size and the same shape. In practice, the substrate will typically consist of particles of varying sizes. Should the particle size variation be small, using an average particle size may provide a sufficiently accurate description of the kinetics of substrate conversion. When the size variation is large, the distribution of particle sizes needs to be taken into account.

We will consider here the distribution of the particle sizes as as a function of the linear particle size x, as used in Equation 8. For simplicity, it is assumed that all particles have the same shape, described by the shape parameter u, with u between 0 and 1 (Equation 3).

The following particle number distribution is a suitable choice from the point of view of both mathematical simplicity and the ability to describe experimental distributions:

(12) $$p_{n}(x)=\frac{\alpha -1}{s^{1-\alpha }}\frac{1}{{\left(x+s\right)}^{\alpha }},$$

**Figure 3:**
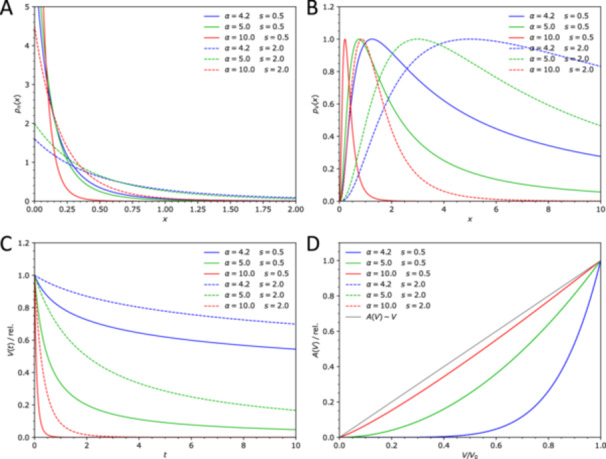
Several examples of distributions of particle sizes showing the effect of the parameters α and s. (A) particle number distributions $p_{n}(x)$ (number of particles of radius x); (B) particle volume distributions $p_{V}(x)$ (total volume of particles of radius x); (C) the remaining total substrate volume V(t) as a function of the reaction time for the distributions shown in (A) and (B); (D) the relationship between the total particle surface area A(V) and the remaining substrate volume V (relative to the initial values) for the distributions shown in A and B.

The two parameters, α and s (s>0), determine the shape of the distribution (Figure 3). The distribution $p_{n}(x)$ is normalized, meaning that its integral over all particle sizes, from x=0 to ∞, is equal to one. A fraction of the number of particles in the interval of sizes between $\left(x_{1},x_{2}\right)$ can obtained by integration of $p_{n}(x)$ over this interval.

Multiplying the particle number distribution $p_{n}(x)$ by the volume V(x) (Equation 9) gives the particle volume distribution $p_{V}(x)\equiv V(x)p_{n}(x)$, which describes the substrate volume of particles of size x. Integration of $p_{V}(x)$ from x=0 to ∞ gives the average particle volume $V_{a}$:

(13) $$V_{a}=\int_{x=0}^{\infty }V(x)p_{n}(x)\mathrm{dx}={\left(\frac{\mathrm{as}}{d}\right)}^{d}\frac{\Gamma (\alpha -d-1)\Gamma (d+1)}{\Gamma (\alpha -1)},$$

where Γ(x) is the gamma function. Because the average particle volume is finite, the values of α are restricted as follows: α>d+1. In general, a smaller α or a larger s means larger contribution of larger particles and a larger average particle volume (Figure 3).

Given the initial particle number distribution $p_{n}(x)$, we can use the linear decrease of the effective radius x derived above (Equation 8) to obtain the expression for the time‐dependent distribution of particle numbers as the reaction proceeds:

(14) $$p_{n}(x,t)=\frac{\alpha -1}{s^{1-\alpha }}\frac{1}{{\left(x+\mathrm{kt}+s\right)}^{\alpha }}.$$

Note, that, because of the dependence on (x+kt), the time dependence of $p_{n}(x,t)$ is effectively a shift of the distribution to the left along the x axis: $p_{n}(x,t)=p_{n}(x+\mathrm{kt},t=0)$. This is true for any distribution, not only that in Equation 12. The integral of $p_{n}(x,t)$ over all particle sizes yields the remaining fraction of the number of particles at any time of the reaction. The temporal evolution of the particle number distribution can also be used to determine the mean effective particle radius $\bar{x}$:

(15) $$\bar{x}\left(t\right)=\frac{\int xp_{n}\left(x,t\right)dx}{\int p_{n}\left(x,t\right)dx}=\frac{s+kt}{\alpha -2}.$$

The mean particle radius increases with time, which is caused by the small particles being consumed fast and the particular size distribution of the remaining particles.

More relevant for practical purposes is the integral of the time‐resolved particle volume distribution $p_{V}(x,t)\equiv V(x)p_{n}(x,t)$, reflecting the remaining volume of the substrate during the reaction:

(16) $$V(t)=\int_{x=0}^{\infty }V(x)p_{n}(x,t)\mathrm{dx}=V_{a}{\left(1+\frac{k}{s}t\right)}^{d-(\alpha -1)}.$$

If the initial substrate volume is denoted $V_{0}$, the factor $V_{a}$ in Equation 16 can be replaced by $V_{0}$ to give directly the remaining substrate volume. This is the main result describing the kinetics of the consumption of substrate consisting of particles with their sizes distributed according to eq. 12.

At this point it is useful to compare the progress of the substrate consumption for the case of a single particle or an ensemble of particles of equal size (Equation 5, for u>1) and the case of particles of different sizes distributed according to Equation 12 (Equation 16). The only difference in the reaction kinetics is in the exponent in the two expressions. The same difference is naturally present in the relationship between the overall reaction rate and the remaining substrate volume, which can be directly obtained from Equation 16 for the size‐distributed substrate:

(17) $$\frac{\mathrm{dV}(t)}{\mathrm{dt}}=\frac{k(d+1-\alpha )V_{a}}{s}{\left(\frac{V(t)}{V_{a}}\right)}^{1+\frac{1}{\alpha -(d+1)}}∼V{\left(t\right)}^{1-\frac{1}{d-(\alpha -1)}}∼V{\left(t\right)}^{u^{\prime }}.$$

This relation can be compared to Equation 4 for a single‐size particle. When the particle sizes are distributed according to Equation 12, the effective particle dimensionality d is replaced by $d^{\prime }=d-(\alpha -1)$, and, consequently, the particle shape parameter u is replaced by an effective shape parameter $u^{\prime }=1-1⁄d^{\prime }$. This rescaling of u fully describes the effect of the particle distribution $p_{n}(x)$ on the reaction kinetics. Because α>d+1, the value of $d^{\prime }$ is negative and therefore $u^{\prime }>1$. This means that the size‐distributed system behaves as a particle with structured, eroded surface (Table 1, Figure 2).

One consequence of the particular form of Equation 16 is that the particle shape (parameter u or, equivalently, d) and the form of the distribution (parameter α) influence the reaction kinetics in the same way. A change of one of the parameters can be therefore exactly compensated for by an appropriate change of the other. This in turn means that the particle shape and the size distribution cannot be determined uniquely from a given reaction course, if no other information is available.

The relationship between the reaction kinetics of a single‐size particles and size‐distributed particles also provides the interpretation of the two parameters, s and α, in the distribution $p_{n}(x)$, beyond their effect on the shape of the distribution (Figure 3). Comparing Equation 10 and eq. 16 shows that s is analogous to the initial particle size $x_{0}$ and directly enters the characteristic rate parameter k/s describing the rate of substrate consumption. Change of s only rescales the time but because it does not affect $u^{\prime }$, it has no influence on the shape of the kinetics.

Conversely, the parameter α defines the rescaling of the shape parameter u to $u^{\prime }$, and in this way influences the shape (temporal profile) of the kinetics (Figure 2): smaller α means stronger reaction slow‐down at later times (large $u^{\prime }$), larger α means that $u^{\prime }$ approaches 1, that is, the reaction approaches exponential kinetics.

### 2.4 Exponential Distribution

Another particle size distribution that is of interest is the exponential distribution. The exponential particle number distribution depends on the reaction time in the following way:

(18) $$p_{n}(x,t)=\frac{1}{s}e^{-\frac{x+\mathrm{kt}}{s}},$$

The only parameter of the distribution is s, and has the meaning of the initial mean particle size. The initial mean particle volume $V_{a}$ is:

(19) $$V_{a}={\left(\frac{\mathrm{as}}{d}\right)}^{d}\frac{\Gamma (d)}{d},$$

The total substrate volume decreases during the reaction exponentially with time:

(20) $$V(t)=\int_{x=0}^{\infty }V(x)p_{n}(x,t)\mathrm{dx}=V_{a}e^{-\frac{k}{s}t},$$

and the overall reaction rate is related to the remaining substrate volume V(t) as:

(21) $$\frac{\mathrm{dV}(t)}{\mathrm{dt}}=-\frac{k}{s}V(t).$$

The reaction rate is directly proportional to the substrate volume V(t), therefore the exponential particle size distribution leads to simple first order kinetics. This is true regardless of the shape of the particles (cylinder, sphere,…), that is, regardless of the particle surface‐to‐volume relationship, as long as its volume V(x) can be expressed by Equation 9 with a constant d. The exponential particle size distribution therefore does not lead to any reaction slowdown. An initially exponential particle size distribution stays exponential during the degradation. The mean particle size ($\bar{x}(t)=s$) and the mean particle volume $\bar{V}(t)=V_{a}$ also remain unchanged during the reaction.

The two introduced particle size distributions (Equations 12 and 18) have the practical advantage that they lead to simple analytical results (Equations 16 and 20), which is important for data analysis. Both also have a useful interpretation, either due to the relation to the effective shape parameter u′ (Equation 12), or due to the resulting first order kinetics (Equation 18).

In practice, a single distribution term may not be sufficient to adequately describe experimental data. Then, a sum (or a difference) of two or more terms as in Equations 12 or 18 may be used to accurately describe the particle size distribution. In the following sections, the particle size distributions are described as a sum of two terms of Equation 12, both with the same particle shape (parameter u). In an even more general model, a combination of two or more terms, each with a different particle shape or a different distribution form, could be used. The particular form of the distribution or its number of parameters are not important, because the model distribution serves only as mathematical parametrization of a real (experimental) particle distribution. The particle distribution $p_{n}(x,t)$ and the substrate volume V(t) are connected via the relation in Equation 16. In principle, one could be calculated from the other one numerically, without the need to parametrize the particle distribution by an analytical function as in Equation 12, although in practice some kind of parametrization or interpolation of the data points would always be performed to facilitate the calculation.

## 3 Application to Experimental Data

### 3.1 Particle Size Distribution Derived From the Reaction Rate Slowdown

**Figure 4:**
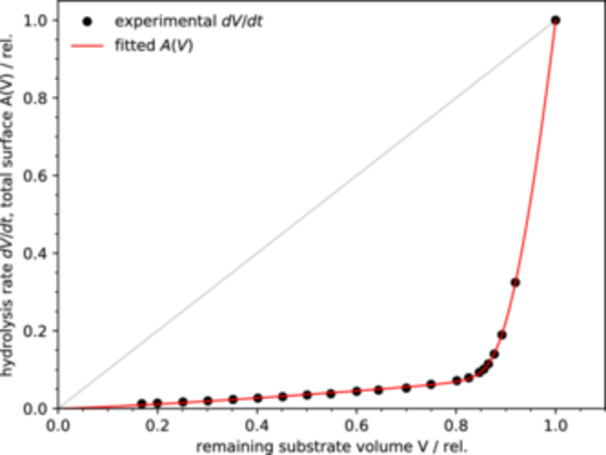
Experimental hydrolysis rate dV/dt and total particle surface area A(V) in dependence on the remaining substrate volume V (all quantities are expressed relative to the initial values). For comparison, a hypothetical hydrolysis rate directly proportional to the remaining amount of substrate is shown as a gray line. The experimental data are take from Ref (Monschein, Reisinger, and Nidetzky 2013).

The rate of enzymatic hydrolysis of cellulosic substrate, such as Avicel, is commonly observed to undergo large changes in the course of the reaction. Often, the rate is initially fast but slows down rapidly after only a small fraction of the substrate is converted, and later decreases further at a lower pace (Monschein, Reisinger, and Nidetzky 2013) (Figure 4).

As an example illustrating this behavior we choose the data of hydrolysis of microcrystalline cellulose Avicel PH‐101 by cellulase mixture from *Trichoderma reesei* (Monschein, Reisinger, and Nidetzky 2013) (Figure 4). The experimentally determined dependence of the hydrolysis rate on the remaining substrate amount shows that the reaction rate decreases rapidly to approximately 10% of its initial value while only about 10% of the substrate is converted. As more substrate is hydrolyzed, the reaction rate decreases gradually further.

An important question is whether, or to what extent, the experimentally observed hydrolysis slowdown (Monschein, Reisinger, and Nidetzky 2013) can be explained as a consequence of changes of the total substrate surface area. The rate of change of the surface area can have its origin in the initial distribution of substrate particle sizes, as described in the previous section. We can further ask what an appropriate particle size distribution would look like.

Comparing the experimental data (Figure 4) with the surface‐volume relationship for particles of different shapes (Figure 2) shows that the significant decrease of the surface area (alternatively, the reaction rate) at low substrate consumption can be expected when the shape parameter u is larger than one, that is, for an eroded particle. As shown in the previous section, this is mathematically equivalent to a size distribution of particles of a simple shape (sphere, cylinder, etc., with u between 0 and 1) described by Equation 12.

**Figure 5:**
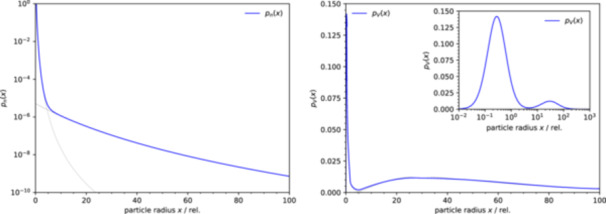
Two representations of the particle size distribution reproducing the experimental hydrolysis rate. Left: the particle number distribution $p_{n}(x)$ (the number of particles of radius x). Right: the particle volume distribution $p_{V}(x)$ (the total volume of particles of radius x); inset: the same distribution on a logarithmic scale.

A single distribution of this type does not, however, fit the experimental data well. Therefore, we tested a two‐component particle number distribution based on Equation 12:

(22) $$p_{n}(x)=c_{1}p_{n}(x,s_{1},\alpha_{1})+c_{2}p_{n}(x,s_{2},\alpha_{2}),$$

and the associated particle volume distribution $p_{V}(x)=V(x)p_{n}(x)$ (Figure 5). It was assumed that both components consist of spherical particles and differ only in the parameters s and α. The factors $c_{1}$ and $c_{2}$ define the contributions of the two components to the overall distribution. Fitting all six parameters results in a very good match with the experimental data (Figure 4). The resulting parameter values are: $s_{1}=0.31$, $s_{2}=45.9$, $\alpha_{1}=6.33$, $\alpha_{2}=7.67$, $c_{1}=5.28$, $c_{2}=3.5\cdot 10^{-5}$. The data contain no time information, therefore the value of the rate constant k was set to 1 in the analysis.

The fitted distribution is clearly bimodal with a small overlap between the two components. The mean particle size of the two components differs by a factor of approximately 110. Although the small‐particle component (index 1) contains many more particles than the large‐particle component (index 2), due to the large particle size difference the large‐particle component accounts for 88% of the total initial substrate volume. The initial sharp decrease of the reaction rate is due to the almost full hydrolysis of the small‐size component. The clear separation of the two components leads to the observed sharp ‘bend’ in the A(V) curve at the point when about 12% of the substrate is consumed (Figure 4).

Although the two‐component distribution describes the data well, there seems to be no obvious reason why the substrate should consist of two distinct groups of particles with such a large average size difference. However, because of the equal effect of the particle size distribution and the shape of the structured particles (u>1) on the overall reaction kinetics, the smaller component can be interpreted as representing a complex surface morphology of large particles, possibly due to much smaller particles adhering to the surface of the larger particles, or simply due to the high porosity of the outer layer of the particles, as can be observed in scanning electron microscopy (SEM) images of Avicel (Hindi 2017). After the initially structured surface of the particles has been consumed by the reaction, the remaining ‘cores’ of the particles are described by the large distribution component.

### 3.2 Reaction Rate Slowdown Derived From the Particle Size Distribution

**Figure 6:**
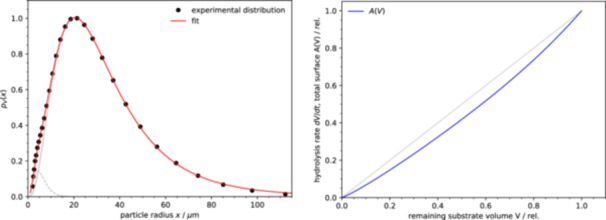
The experimental particle size distribution and its calculated effect on the degradation rate. Left: the experimental particle volume distribution $p_{V}(x)$ (total volume of particles of radius x) fitted to a two‐component model (gray curves: the two components plotted separately). Right: the total particle surface area A(V), proportional to the reaction rate dV/dt, in dependence on the remaining substrate volume V derived from the particle size distribution shown on the left. The gray line is a hypothetical reaction rate directly proportional to the remaining amount of substrate (all quantities are expressed relative to the initial values).

In a second example we applied the introduced formalism to deduce the reaction rate using an experimentally determined particle size distribution. We chose the distribution of the particle sizes of Avicel PH‐101 that has been previously determined by laser diffraction (Pharmahub 2022) (Figure 6A). This experimental distribution can be approximated very well by the two‐component model as used above (Equation 22). The parameters of the fit are: $s_{1}=188$, $s_{2}=58.6$, $\alpha_{1}=134.9$, $\alpha_{2}=11.59$, $c_{1}=0.0127$, $c_{2}=0.00497$. In this case, and contrary to the previous example, the two components overlap strongly, with the larger component (index 2) accounting for 98% of the total particle volume.

Assuming that the degradation rate is proportional to the total surface area A we can calculate (Equations 22 and 16) the dependence of the reaction rate on the remaining substrate volume V (Figure 6). The resulting reaction rate slowdown is weak, not far from the first‐order kinetics, and is rather different form the experimental slowdown shown above (Figure 4).

At first sight, this difference seems to lead to the conclusion that the surface area changes during the degradation are not sufficient to explain the observed reaction slowdown. However, there are several reasons to assume that the experimental particle size distribution does not accurately reflect the substrate morphology. The data cover only the size range of two orders of magnitude (here approximately 1–100 μm); particles outside this range are not accounted for correctly. This may be an important limitation, considering that in the previous example the size distribution spans more than three orders of magnitude (Figure 5). The particle size at the maximum of the small‐size component in Figure 5 is approximately 110 times smaller than that of the large‐size component. To have a similar effect on the kinetics, in analogy with the distribution in Figure 5, the experimental distribution in Figure 6 with a maximum of the major component at 20 μm would have to additionally contain a small‐size component with a maximum at approximately 0.2 μm. A higher resolution capable of detecting smaller particles would therefore be required to confirm if such a small‐size component is present.

Furthermore, small particles may be aggregated or adhering to the surface of large particles, thus forming a highly structured, porous particle surface. The aggregated structure and the high porosity are well documented properties of Avicel particles (Doelker 1993; Thoorens et al. 2014), and have been directly visualized by SEM in case of Avicel (Hindi 2017; Thoorens et al. 2014) and also other cellulosic substrates (Piątek, Lisowski, and Dąbrowska 2021; Tobyn 1998; Vallejo et al. 2021). Loosely aggregated particles may act as a single larger solid particle in laser diffraction, and thus will not be correctly represented in the experimental particle size distribution.

To correctly asses the contribution of small, possibly aggregated particles responsible for the fast initial phase of the reaction kinetics, methods are required that could be applied to particles in an aggregated state. The quantification of the structural detail of cellulosic substrate below the optical resolution has been attempted by transmission electron microscopy (TEM) in combination with optical imaging (Olsen et al. 2016). The estimated surface area was found to positively correlate with the reaction rate. Although an accurate quantification of TEM images is challenging, this approach is promising and could provide the missing structural data in the sub‐μm spatial range.

## 4 Conclusion

In this work, a general framework to describe the effects of the shape of the substrate particle and the particle size distribution on the rate of surface‐limited reactions is introduced.

It is shown that a single numerical parameter u accounts for the effects of a broad range of substrate shapes. This parameter describes not only simple shapes like a sphere or a cylinder, but also more complex substrates, such as crystals with enzyme binding sites only on a part of the surface, where the available surface area increases as the reaction proceeds. It can be also applied to particles with structured surface, thus modeling the surface of pre‐processed substrate.

For a particular type of size distribution (Equation 12) the effect of the distributed particle size is found to be mathematically equivalent to the effect of the shape of a particle with structured, that is, eroded or porous surface, described by the shape parameter u larger than one. This fact can potentially complicate the interpretation of kinetics data because these two effects cannot be easily separated from each other based only on the reaction rate.

This apparent ambiguity between the particle shape and the size distribution can be rationalized by comparing two hypothetical substrates: one consists of particles of a simple shape (e.g., spherical) with a broad size distribution. The second substrate is obtained by a thought transformation of the first substrate by letting the smaller particles adsorb onto the surface of the larger particles, resulting in compound particles of similar size with a structured, porous surface (u>1). As long as the resulting aggregates are sufficiently porous so that a large surface fraction of the aggregated particles is still accessible to the enzyme, the reaction kinetics of the two substrates will remain similar (see also the illustration of the case u>1 in Figure 1).

The presented models of particle shape and size distribution lead to analytical expressions for the reaction kinetics, which is particularly convenient for fast and efficient analysis of experimental data. More general particle size distributions can be expressed as a combination of several simple distributions (e.g., as in Equation 22) while maintaining the advantage of the analytical solution.

The application of the presented formalism to the experimental data revealed that a particle size distribution with a significant volume of small particles is required to explain the observed slowdown of the reaction kinetics. When the experimental size distribution does not capture this small‐size component, the predicted reaction kinetics exhibits only a mild rate slowdown. As very small particles and the fine surface structure, porosity or aggregation state of the larger substrate particles may not be correctly detected by optical methods, techniques with higher spatial resolution, such as TEM, are required to characterize the substrate in a sufficient detail.

It can be concluded that the distribution of particle sizes and the change of the overall surface‐to‐volume ratio during the reaction can result in an effect that is sufficiently strong to account for the degradation rate slowdown. To ascertain experimentally to which extent this is the actual cause of the observed slowdown, not only the particle size but also the particle aggregation state affecting the surface morphology and the internal pore structure needs to be quantified.

The presented results are general in a sense that no specific features of the reaction mechanism of the enzyme, the type of bond fission (hydrolytic, oxidative, etc.), or any particular properties of the substrate material, were considered. They are, in principle, applicable to any reaction mechanism and any type of substrate material, whether of biological origin or synthetic. In reality, the particle shape and its size are not the only factors determining the reaction kinetics; other aspects of the enzyme‐solid substrate interactions have been implicated previously: stalled processive enzymes (Yang, Willies, and Wyman 2006), enzyme blocking by other obstacles (Kafle et al. 2015; Yu et al. 2012), nonproductive binding to lignin in lignocellulosic feedstocks or to other structures (Palonen et al. 2004; Berlin et al. 2005; South, Hogsett, and Lynd 1995; Nutor and Converse 1991), synergy effects between cellulases (Ganner et al. 2012), substrate heterogeneity, enzyme crowding (Väljamäe et al. 1998; Igarashi et al. 2011; Levine et al. 2010), and so forth. The introduced formalism can be combined with these additional effects to formulate more comprehensive models.

## Author Contributions

**Zdeněk Petrášek:** conceptualization, methodology, validation, writing–original draft, review and editing. **Bernd Nidetzky:** conceptualization, supervision, writing–review and editing.

## Data Availability

The data that support the findings of this study are available from the corresponding author upon reasonable request.
